# Bcl6 Is Required for Somatic Hypermutation and Gene Conversion in Chicken DT40 Cells

**DOI:** 10.1371/journal.pone.0149146

**Published:** 2016-02-22

**Authors:** Alan M. Williams, Yaakov Maman, Jukka Alinikula, David G. Schatz

**Affiliations:** 1 Department of Immunobiology, Yale University School of Medicine, New Haven, Connecticut, United States of America; 2 Howard Hughes Medical Institute, Chevy Chase, Maryland, United States of America; Michigan State University, UNITED STATES

## Abstract

The activation induced cytosine deaminase (AID) mediates diversification of B cell immunoglobulin genes by the three distinct yet related processes of somatic hypermutation (SHM), class switch recombination (CSR), and gene conversion (GCV). SHM occurs in germinal center B cells, and the transcription factor Bcl6 is a key regulator of the germinal center B cell gene expression program, including expression of AID. To test the hypothesis that Bcl6 function is important for the process of SHM, we compared WT chicken DT40 B cells, which constitutively perform SHM/GCV, to their Bcl6-deficient counterparts. We found that Bcl6-deficient DT40 cells were unable to perform SHM and GCV despite enforced high level expression of AID and substantial levels of AID in the nucleus of the cells. To gain mechanistic insight into the GCV/SHM dependency on Bcl6, transcriptional features of a highly expressed SHM target gene were analyzed in Bcl6-sufficient and -deficient DT40 cells. No defect was observed in the accumulation of single stranded DNA in the target gene as a result of Bcl6 deficiency. In contrast, association of Spt5, an RNA polymerase II (Pol II) and AID binding factor, was strongly reduced at the target gene body relative to the transcription start site in Bcl6-deficient cells as compared to WT cells. However, partial reconstitution of Bcl6 function substantially reconstituted Spt5 association with the target gene body but did not restore detectable SHM. Our observations suggest that in the absence of Bcl6, Spt5 fails to associate efficiently with Pol II at SHM targets, perhaps precluding robust AID action on the SHM target DNA. Our data also suggest, however, that Spt5 binding is not sufficient for SHM of a target gene even in DT40 cells with strong expression of AID.

## Introduction

While V(D)J recombination is the principle means to generate a broad primary antibody repertoire in most species, there are three additional immunoglobulin (Ig) gene diversification processes which are dependent on the activation induced cytosine deaminase (AID). AID deaminates cytosine residues in single-stranded DNA creating U:G mismatches that can be converted into mutations and DNA breaks during gene conversion (GCV), somatic hypermutation (SHM), class switch recombination (CSR) [1]. In GCV, donor DNA sequences serve as templates to be copied into the rearranged variable (V) region [2]. GCV has been best characterized at the chicken Ig light chain (*IgL*) locus with 25 pseudo V genes upstream of the *IgL* V region serving as GCV donor sequences for the single rearranged VJ element [3]. SHM introduces point mutations at rearranged V regions and typically occurs in the context of an immune response. SHM rates greatly exceed background mutation levels throughout the genome and, when combined with selection mechanisms, serves as the basis for affinity maturation [4]. Finally, CSR involves DNA breaks in switch regions to replace one set of Ig heavy chain constant region exons with another thereby altering the antibody isotype [1].

During an immune response, antigen engaged B cells can form germinal centers (GCs), which are the classical sites of SHM and CSR in secondary lymphoid organs. Consistent with the Ig diversification taking place, GC B cells express the highest levels of AID [5] and are tightly regulated via multiple B cell gene expression pathways and cellular interactions [6]. Bcl6 is required for the formation and maintenance of the GC reaction [6] and is a key regulator of the GC B cell gene expression program, modulating the expression of genes involved in GC B cell differentiation, cell cycle regulation, and maintenance of the GC B cell phenotype [7–9]. For example, Bcl6 represses expression of *Prdm1*, thereby helping to prevent the differentiation of GC B cells into Ig secreting plasmablasts [8, 10, 11]. In addition, Bcl6 suppresses the induction of a robust DNA damage response, thereby helping to keep GC B cells alive during the genotoxic processes of SHM and CSR. The B cell does, however, place limits on tolerable levels of DNA damage as Bcl6 degradation occurs in response to excessive genotoxic stress with a concurrent induction of apoptosis [12]. Thus, Bcl6 is intimately involved in the regulation of germinal center physiology.

Growing evidence suggests that AID targeting to and activity at Ig genes is strongly linked to transcription by RNA polymerase II (Pol II). Transcription of the target substrate is required for SHM [13, 14], and AID activity can be correlated with transcriptional intensity [14]. However, transcription alone cannot explain SHM and CSR since many genes are transcribed in GC B cells and are either not targeted by AID at all or not at the level observed at the Ig loci [15]. The IgV promoter is not a strict requirement, as heterologous promoters potentiate strong V region SHM. Furthermore, the V region itself is not unique *per se* as heterologous sequences replacing the V region can be targeted for mutation [16, 17]. Despite being part of the same transcription unit as the V exon, the constant region exons undergo no SHM [18], underscoring the fact that SHM is exquisitely regulated and targeted.

Many studies have attempted to define the specific transcriptional regulatory mechanisms that potentiate SHM. During the initiation phase of transcription, the C-terminal domain of Pol II is hypophosphorylated upon intital recruitment to the promoter, where, during promoter clearance, it undergoes phosphorylation at serine 5 (pSer5 Pol II) [19]. pSer5 Pol II complexes accumulate ~40 nucleotides downstream of the transcription start site, in part due to their association with the negative elongation factor (NELF) and the DRB-sensitivity inducing complex (DSIF), which is composed of Spt4 and Spt5 [20]. The release of the paused pSer5 Pol II complex into elongation mode occurs following an additional phosphorylation event on serine 2 of the Pol II C-terminal domain and phosphorylation of DSIF and NELF, with NELF dissociating from and DSIF remaining associated with elongating Pol II [21, 22]. Interestingly, studies have demonstrated that Spt5, which can induce pausing or stalling of Pol II *in vitro*, [23, 24] interacts directly or indirectly with AID [25]. The importance of this interaction was supported by the finding that, in *ex vivo* activated B cells overexpressing AID, the genes that robustly recruited Spt5 also underwent SHM [25]. Furthermore, it was found that Spt5 recruitment was directly proportional to promoter-proximal Pol II stalling at genes genome-wide, with the Ig heavy chain (*IgH*) locus defined as the strongest recruiter of Spt5 in the B cell genome [25]. These observations suggest that Spt5, through its interaction with AID and stalled Pol II, could provide AID prolonged access to DNA, thereby permitting efficient deamination.

To investigate the potential link between Bcl6 and the molecular mechanism of SHM, we compared Bcl6-sufficient and -deficient DT40 cells. We found that robust expression of AID did not restore GCV/SHM of the IgL V region in Bcl6-deficient DT40 cells, nor could it restore SHM of a *GFP* substrate flanked by a potent SHM targeting element. Several known parameters of SHM including substrate transcription levels, single-stranded DNA content of the substrate, and AID nuclear localization, were not substantially altered by the absence of Bcl6. In contrast, binding of Spt5 in the body of a SHM-targeted *GFP* gene was substantially reduced relative to levels at the promoter in the absence of Bcl6, while partial Bcl6-reconstitution largely restored Spt5 binding but not SHM. These data suggest one role of Bcl6 is to promote Spt5 association with SHM targets, providing a partial explanation for the defect in SHM in the absence of Bcl6.

## Results

### AID does not restore SHM and GCV in Bcl6-deficient DT40 cells

Upon deletion of Bcl6, DT40 cells lose expression of AID and UNG mRNA [26] and hence would be expected to lack SHM and GCV activity. It is unknown if re-expression of AID in a Bcl6-deficient setting would permit SHM and GCV. To test this, we infected Bcl6-deficient DT40 cells with a retroviral vector expressing chicken AID and a thy 1.1 surface marker, and after growing the cells for 28 days, sequenced the IgL V region to detect point mutations and gene conversion events. Strikingly, no mutation events could be detected in Bcl6^-/-^ AID^R^ DT40 cells, yielding a mutation event frequency (a combination of SHM and GCV events) lower than that of Bcl6^-/-^ thy1.1 cells that lack detectable AID expression (data not shown). These data suggest that re-expression of AID in Bcl6^-/-^ DT40 cells is not sufficient to restore SHM or GCV. It is important to note, however, that Bcl6^-/-^ DT40 cells are substantially compromised in their growth rate, with subclones becoming visible by eye at much later time points after subcloning (14–21 days) than is the case for WT DT40 cells (8–10 days) (data not shown). Hence, the finding that Bcl6^-/-^ DT40 cells expressing substantial amounts of AID do not perform SHM or GCV should be interpreted cautiously.

### Pax5 cannot induce SHM and GCV in Bcl6^-/-^ cells

It was previously shown that expression of the transcription factor Pax5 is lost upon deletion of Bcl6 in DT40 cells and that forced expression of Pax5 in these cells is not sufficient to restore AID expression [26]. We obtained Bcl6^-/-^ DT40 cells reconstituted with Pax5 (Bcl6^-/-^ Pax5^R^) [26] and found that Pax5 re-expression dramatically improves the growth rate of Bcl6^-/-^ cells, with subclones becoming visible 9–12 days after subcloning (data not shown). Hence, subsequent mutation analyses were performed in Bcl6^-/-^ Pax5^R^ cells.

**Fig 1:**
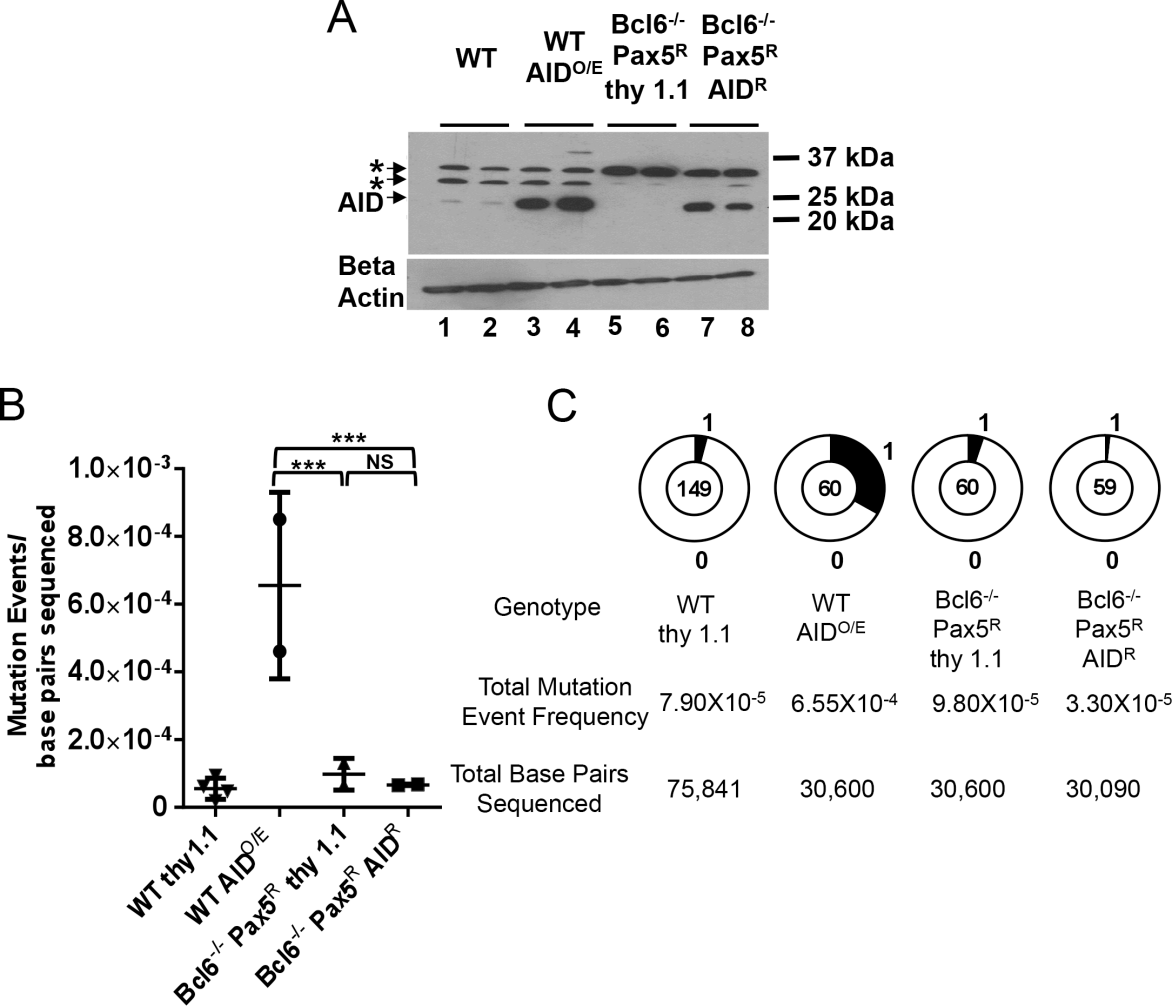
Pax5 expression does not restore SHM/GCV in Bcl6-deficient DT40 cells. A) Western blot analysis for AID expression in the subclones analyzed for mutations in panels B and C with a beta actin loading control shown below. *, background bands. B) Scatter plot of IgL V region SHM/GCV event frequencies from individual subclones as indicated. Horizontal bars indicate the mean frequency of mutations for each genotype. Z test used to assess significant differences among mutation frequencies. ***, p<0.001 and NS, not statistically significant. C) Pie charts showing the distribution of all SHM/GCV events observed at the IgL V region from the independent subclones indicated in panel B after 28 days of culture.

Bcl6^-/-^Pax5^R^ cells were infected with AID-expressing and control retroviruses and Western blotting confirmed that Bcl6^-/-^ Pax5^R^ AID^R^ cells express AID while Bcl6^-/-^ Pax5^R^ thy1.1 cells do not (Fig 1A, lanes 5–8). WT cells were infected with the same viruses to yield WT AID^O/E^ and WT thy1.1 lines, with the former expressing much higher levels of AID than the latter (Fig 1A, lanes 1–4).

The cell lines that were analyzed in Fig 1A were subcloned, grown for 28 days, and their IgL V regions were sequenced for SHM and GCV events. Bcl6^-/-^ Pax5^R^ AID^R^ DT40 cells displayed a mutation event frequency lower than the background established by their Bcl6^-/-^Pax5^R^ thy1.1 (AID non-expressing) counterparts (Fig 1B and 1C). In contrast, WT AID^O/E^ cells mutated at a significantly higher frequency, 6.5 fold greater than the Bcl6^-/-^Pax5^R^ thy1.1 cells (Fig 1B and 1C). These data strongly suggest that deficiency in Bcl6 prevents V region SHM and GCV in DT40 cells, even when they express high levels of AID, and that enforced expression of Pax5 is not sufficient to overcome this defect. We note that a lack of GCV events in Bcl6-deficient cells is not surprising given their poor Ung expression and the importance of Ung for GCV [27, 28]. However, the absence of Ung expression in these cells should have led to an increased susceptibility to AID-dependent point mutations [27]; the lack of such mutations in the Bcl6^-/-^Pax5^R^ thy1.1 cells argues strongly that AID is not acting on the V region in these cells.

### GFP SHM reporter assay

In an attempt to establish a more rapid method of detecting SHM in Bcl6-deficient DT40 cells, we turned to the GFP2 reporter assay, in which SHM is measured by the frequency at which cells expressing green fluorescence protein (GFP) from a strong viral promoter lose GFP fluorescence [29]. Our previous studies demonstrated that GFP loss is greatly accelerated by the presence of DIVAC (Diversification Activator) sequences adjacent to the GFP transcription unit, and that DIVAC is composed of Ig enhancer and enhancer-like sequences that depend on multiple transcription factor binding motifs for their activity [16, 29–31]. Prior studies also demonstrated that GFP loss is AID-dependent and provides an accurate measure of mutation accumulation in the *GFP* gene, and that DIVAC dramatically stimulates SHM while increasing levels of *GFP* transcription less than two fold [16, 29–31].

**Fig 2:**
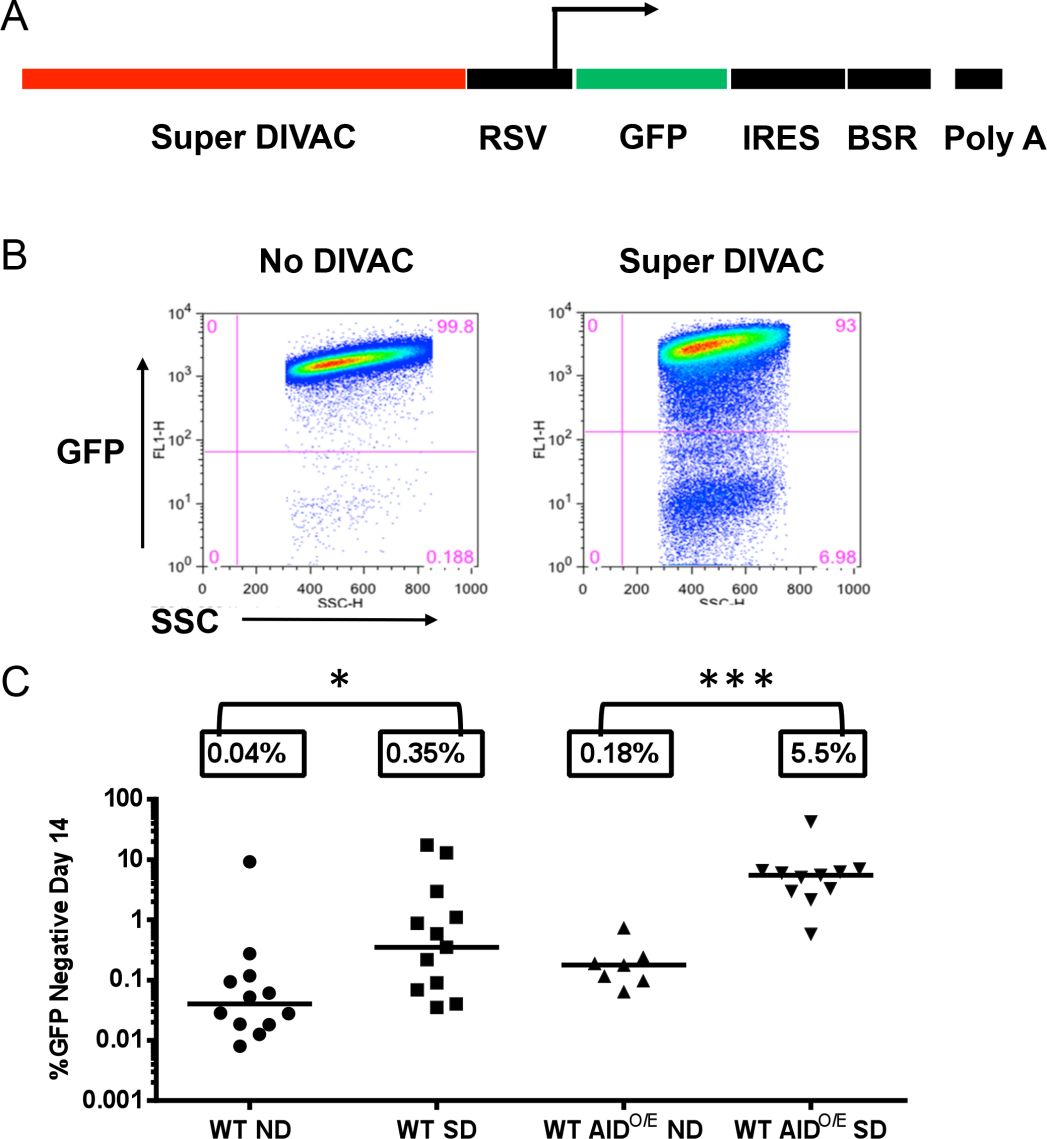
The Super DIVAC GFP2 reporter. A) Schematic diagram of the Super DIVAC GFP2 reporter plasmid (5' to 3'): Super DIVAC in red, Rous sarcoma virus (RSV) promoter in black, GFP coding region in green, and an internal ribosome entry site (IRES), blasticidin resistance gene, and SV40 polyadenylation signal in black. An arrow indicates the approximate location of the transcription start site. B) Representative flow cytometry plots of a No DIVAC and Super DIVAC GFP2 transfected sublcone (over-expressing AID). GFP negative gate was drawn one log below the major GFP positive population. C) Scatter plot of observed percentage of GFP negative cells after 14 days of culture. 7–12 subclones were analyzed for each cell line. Horizontal bars and boxed percentages indicate the median GFP loss frequencies. Mann-Whitney test used to compare median GFP loss values. *, p<0.05 and ***, p<0.001. ND, no DIVAC and SD, Super DIVAC.

To create a strong DIVAC element, we assembled a 2.2 kb fragment (referred to as Super DIVAC) from three previously documented fragments with DIVAC activity: the human Ig lambda enhancer [30], the chicken IgL 5' and 3' core regions with their intervening sequence (which derive from the region downstream of the IgL constant region) [16], and the human IgH intronic enhancer [30] (in order 5' to 3'). Super DIVAC was inserted upstream of the GFP2 cassette (Fig 2A) and constructs containing or lacking Super DIVAC were transfected into the WT DT40 cells from which the Bcl6-deficient DT40 lines were derived. All substrate integrations into the genome were random. The transfected cells either relied on endogenous levels of AID expression or overexpression of AID from the thy1.1 retrovirus (WT AID^O/E^). Transfected cells were subcloned and GFP fluorescence intensity was assessed via flow cytometry after 14 days of culture, with representative GFP flow cytometry results shown in Fig 2B.

In WT DT40 cells, Super DIVAC stimulated GFP loss nearly nine fold above the no DIVAC control (Fig 2C). In WT AID^O/E^ cells, the background of GFP loss in the absence of DIVAC was about five fold greater than in WT DT40 cells, but the stimulation afforded by DIVAC increased to 30 fold (Fig 2C). Hence, Super DIVAC can stimulate SHM when AID expression is limiting, and strongly stimulate SHM when AID levels are high, consistent with previous findings [16, 29–31]. These data indicate that GFP loss is dependent on both AID and Super DIVAC and that the Super DIVAC GFP2 reporter assay is appropriately regulated in the WT DT40 cells used here.

### Abnormal GFP regulation in the absence of Bcl6

**Fig 3:**
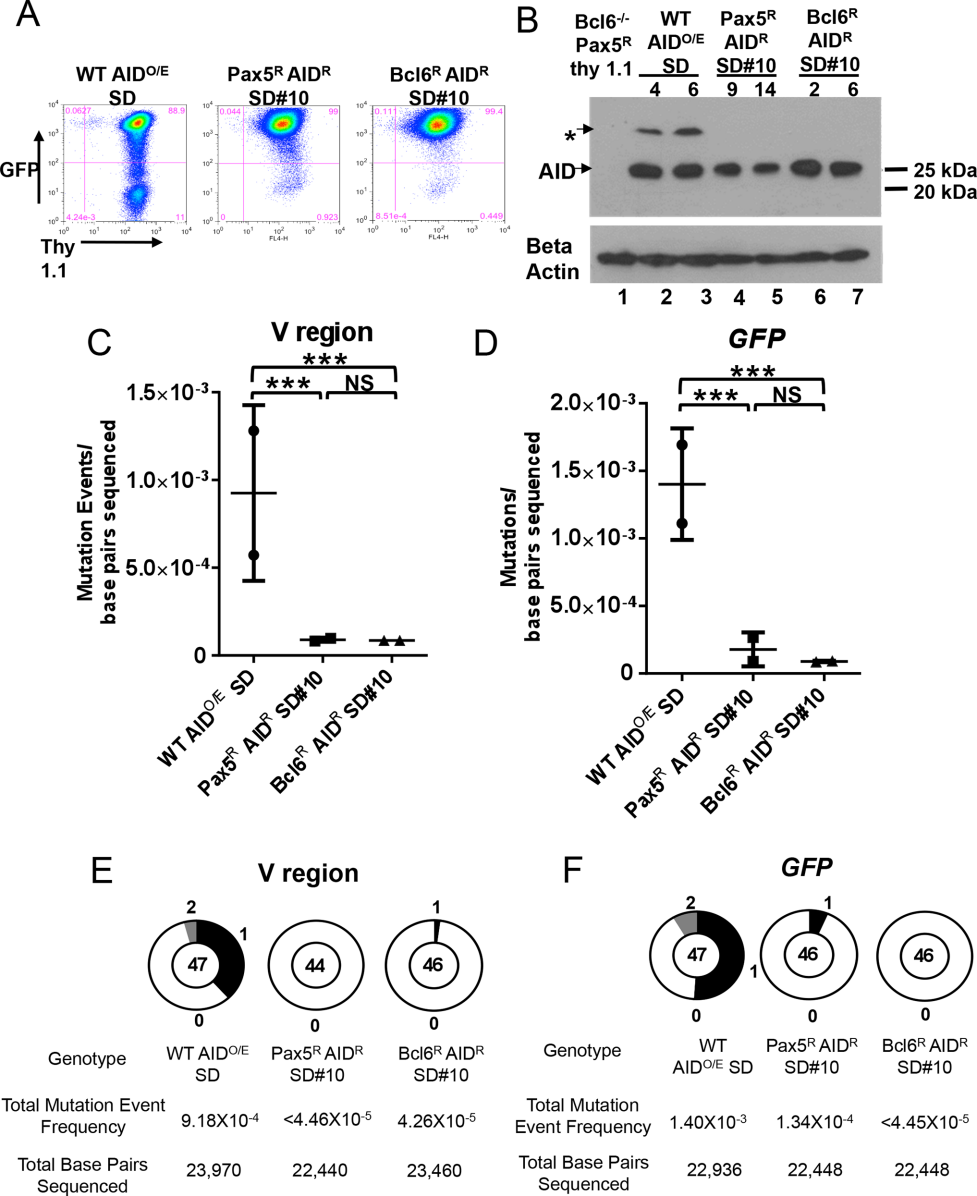
Super DIVAC does not potentiate mutation in the absence of Bcl6. A) Representative flow cytometry plots of GFP and Thy 1.1 levels after 28 days of culture of the indicated cell lines. B) Western blot analysis for AID expression in the sublcones analyzed for mutation in panels C, D, E, and F, with beta actin loading control shown below. * background band. Each WT subclone contains a unique Super DIVAC GFP2 reporter integration event while all Pax5^R^ AID^R^ SD#10 and Bcl6^R^ AID^R^ SD#10 subclones share an identical reporter integration site. C, D) Scatter plots showing the SHM/GCV event frequencies observed at the IgL V region (C) or *GFP* (D) for 2 independent subclones as indicated after 76 days of culture, presented as in Fig 1B. Horizontal bars indicate the mean frequency of mutations for each genotype. E, F) Pie charts showing the distribution of all SHM/GCV events observed at the IgL V region (E) or *GFP* (F) compiled from the 2 independent subclones in panels C, D after 76 days of culture, presented as in Fig 1C. Z test was used to assess significance differences among mutation frequencies. ***, p<0.001 and NS, not statistically significant.

The no DIVAC or Super DIVAC GFP2 reporter plasmids were transfected into Bcl6^-/-^Pax5^R^ DT40 cells and single cell subclones were derived. Surprisingly, after short-term culture, many clones exhibited substantial GFP loss in the apparent absence of AID (data not shown) at frequencies well above the background established from published AID deficient controls (0.006%) [16]. The GFP loss was not dependent on Super DIVAC, as the no DIVAC clones also demonstrated high frequencies of GFP negative cells (data not shown). As detailed below, GFP loss in these cells is not due to mutation accumulation in GFP. Hence, the GFP2 assay does not provide a reliable proxy for SHM in the Bcl6^-/-^Pax5^R^ cells. While it is not clear what causes the instability of GFP in Bcl6-deficient cells, poor *GFP* transcription *per se* is unlikely to be the cause. One Super DIVAC GFP2 clone (SD#10) with robust GFP expression, comparable to that seen in WT cells containing the same construct, was identified. GFP fluorescence levels in the major GFP high population in this integrate was comparable to that in WT clones (e.g., compare plots in Fig 3A). Analyses of transcript levels from subclones derived from the SD#10 parental cell line also indicated robust *GFP* transcription (see below). While we cannot rule out reduced *GFP* transcription in some cells, these data demonstrate that substantial transcription of *GFP* was occurring in the Bcl6^-/-^Pax5^R^ SD#10 background, and so we used the Bcl6^-/-^Pax5^R^ SD#10 cells for the remainder of the SHM and GCV analyses.

### Attempts to reconstitute Bcl6 expression in Bcl6^-/-^Pax5^R^ cells

To attempt to complement the defect in SHM and GCV in Bcl6^-/-^Pax5^R^ cells, Bcl6 was re-expressed in Bcl6^-/-^ Pax5^R^ SD#10 cells using a combination of a standard transfected expression vector (identical to that used previously [26] and a retroviral expression vector, yielding Bcl6^-/-^ Pax5^R^ Bcl6^R^ SD#10 (abbreviated as Bcl6^R^ SD#10). Use of the two expression vectors together yielded improved Bcl6 reconstitution compared to either of them individually, as assessed by changes in the expression of Bcl6 target genes (data now shown and see below).

We used sequencing to determine whether Bcl6^R^ SD#10 cells had regained GCV/SHM of the IgL V region, and whether the V region mutation defect seen in Bcl6-deficient cells extended to the Super DIVAC GFP2 reporter construct. Three lines were studied: WT AID^O/E^ cells containing Super DIVAC GFP2 (WT AID^O/E^ SD), Bcl6^-/-^ Pax5^R^ SD#10 cells reconstituted with the AID-expressing retrovirus (Pax5^R^ AID^R^ SD#10) and Bcl6^R^ SD#10 cells infected with the AID-expressing retrovirus (Bcl6^R^ AID^R^ SD#10). Two subclones of each line were grown for an extended period of 76 days to increase the sensitivity of the assay, and the *GFP* gene and IgL V region were sequenced. Strong AID expression was confirmed in the sequenced subclones by western blot analysis (Fig 3B).

Strikingly, virtually no mutations were detected at the V region (Fig 3C and 3E) or at *GFP* (Fig 3D and 3F) in the Pax5^R^ AID^R^ SD#10 subclones, while mutation events were readily detectable in the WT AID^O/E^ SD subclones at both regions, at frequencies approximately 10 fold higher than in the Pax5^R^ AID^R^ SD#10 subclones. These data demonstrate that in the absence of Bcl6, robust AID expression is not sufficient to restore SHM and GCV at the endogenous IgL V region or SHM at a heterologous mutation substrate, suggesting that Bcl6 potentiates mutation through a general mechanism that is not specific to the *IgL* promoter or V region. We cannot rule out the possibility that the remaining growth defect or reduced AID expression (Fig 3B) in the Pax5 reconstituted cells combine to reduce SHM/GCV. However, the Bcl6 reconstituted cells demonstrated AID levels comparable to WT (Fig 3B), have strongly reduced *Ung* expression (S1C Fig) which enhances SHM activity [27], and still underwent no mutation above background during the 76 days of culture, suggesting that AID and growth rates were not the limiting factors for SHM/GCV in the absence of Bcl6. Surprisingly, the Bcl6^R^ AID^R^ SD#10 subclones also exhibited almost no mutation activity at the V or *GFP* genes (Fig 3C–3E) which prompted us to investigate the effectiveness of the Bcl6 reconstitution.

### Assessing the Bcl6 reconstitution

The fact that the Bcl6^R^ AID^R^ SD#10 subclones did not mutate to any appreciable extent was an unexpected finding and called into question the efficacy of the Bcl6 reconstitution. To address this issue, the Bcl6^R^ AID^R^ SD#10 line was analyzed for evidence of Bcl6 mRNA and protein expression. RT-PCR analysis revealed that Bcl6 mRNA levels were approximately 3.5 fold higher in Bcl6^R^ AID^R^ SD#10 cells than in WT AID^O/E^ SD cells, while no signal was detected for the Pax5^R^ AID^R^ SD#10 line as expected (S1A Fig). Therefore, there is no defect in Bcl6 mRNA expression in the Bcl6^R^ AID^R^ SD#10 sequenced cells.

Bcl6 protein expression was analyzed by Western blot in cytoplasmic and nuclear fractions from the Bcl6-deficient and -reconstituted sequenced lines, taking advantage of a triple Flag tag present at the N-terminus of the chicken Bcl6 protein expressed from the two expression vectors. A distinct band was detected in the nuclear fraction from Bcl6^R^ AID^R^ SD#10 cells at a position consistent with an approximately 100 kDa size for 3X Flag-tagged Bcl6 (S1B Fig). No such band could be detected in either the WT AID^O/E^ SD or Pax5^R^ AID^R^ SD#10 lines. We conclude that Bcl6 protein is expressed to some extent in the Bcl6^R^ AID^R^ SD#10 cells. However, it is not clear how this protein expression level compares to WT levels because we have not found a commercially available antibody that recognizes chicken Bcl6 with which to make a direct comparison.

With evidence for Bcl6 mRNA and protein expression, we analyzed five genes whose expression was known to be altered by the loss of Bcl6 [26] to determine if these loci had responded to the re-expression of Bcl6. For consistency, the lines used in this RT-PCR analysis were those sequenced in Fig 3 and, for comparison, unmanipulated Bcl6^-/-^ cells were also analyzed. We found that Pax5 reconstitution was insufficient to fully restore WT expression levels among the five analyzed genes (S1C and S1G Fig), yet Bcl6 reconstitution altered expression levels for *Mitf*, *Bach2*, and *Aicda* such that they were no longer significantly different from WT cells (S1B, S1C–S1E Fig). We note that *Ung* and *Prdm1* expression were largely unaffected by the Bcl6 reconstitution and remained significantly different from WT expression levels (S1A and S1D Fig). Taken together, the gene expression data indicate that the Bcl6 reconstitution we were able to achieve was partial and was insufficient to restore a WT gene expression program. Particularly notable was the inability of Bcl6 reconstitution to fully suppress *Blimp1* expression, and the fact that the reconstituted cells did not regain the ability to perform GCV/SHM (Fig 3C and 3D). We note that previous work with this same Bcl6^-/-^ DT40 line achieved one clone with robust Bcl6 reconstitution that restored the WT gene expression program [26]. Hence, the Bcl6-deficient cells are unlikely to be intrinsically unable to regain GCV/SHM activity, but rather, a successful Bcl6 reconstitution might be a relatively rare event. We comment on possible reasons why this might be the case in the *Discussion*.

### Analysis of mechanisms by which Bcl6 contributes to GCV/SHM

**Fig 4:**
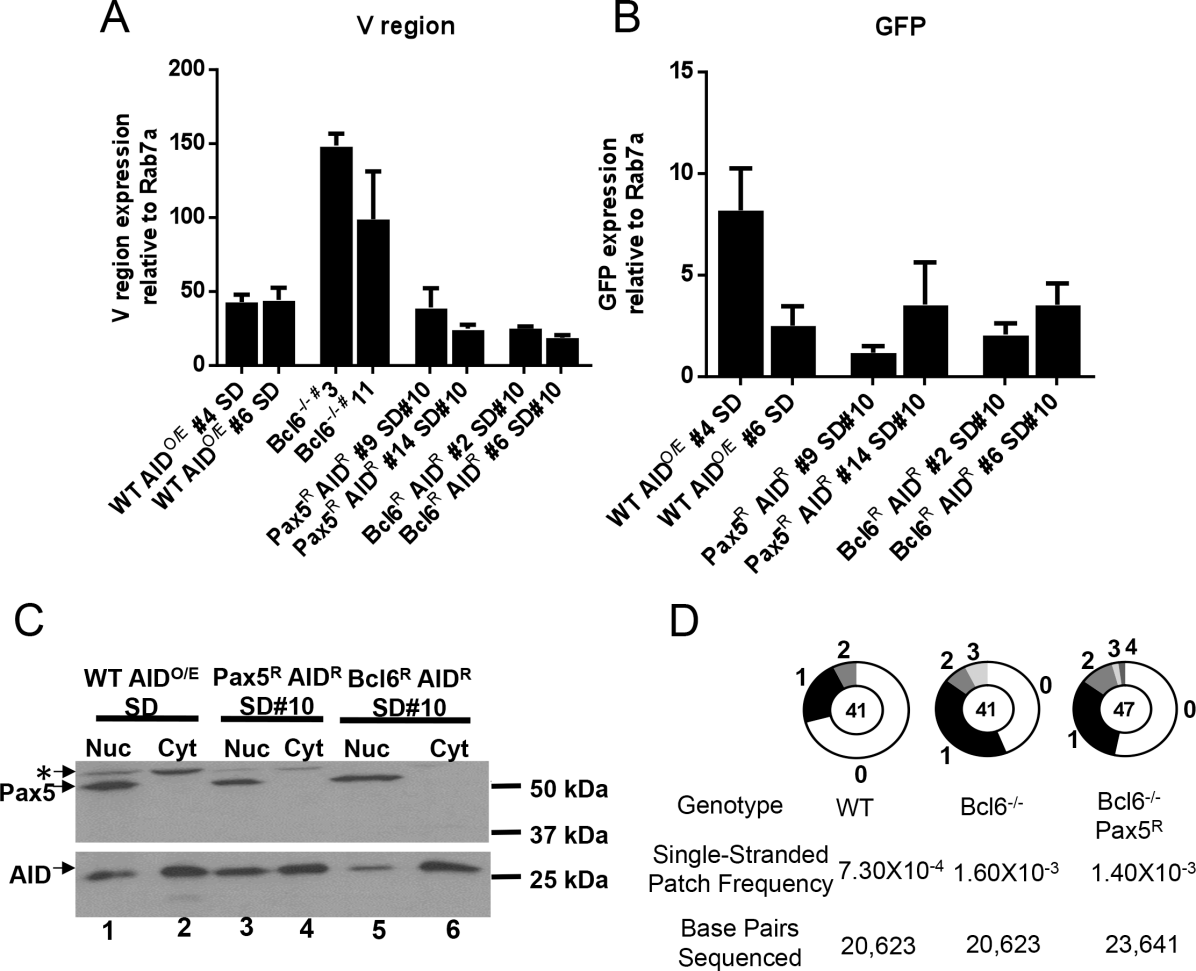
Transcription, AID subcellular localization, and single stranded DNA in Bcl6-deficient DT40 cells. A, B) RT-PCR analysis of A) IgL V region or B) GFP RNA levels in the sequenced subclones from Fig 3C–3F. Data are presented as the average of the signal obtained from two independent RNA preparations for each subclone after normalization to the signal obtained for Rab7a. Error bars represent the SEM. C) Western blot analysis for AID (lower panel) and Pax5 (upper panel) protein in cytoplasmic and nuclear fractions for the indicated cell lines. All fractions were quantitated and equivalent amounts of protein were loaded. *, unknown band. D) Pie charts showing the distribution of single stranded patches detected in the IgL V region after bisulfite treatment and sequence analysis for one subclone of each genotype. Bisulfite converts dCs on exposed single-stranded DNA to dUs and, after PCR amplification and sequencing, C to T transitions and G to A transitions reveal single-stranded dCs on the top and bottom strands, respectively. To be counted as a single-stranded DNA patch, at least two successive transition mutations were required.

To attempt to gain mechanistic insight into how Bcl6 potentiates SHM, we systematically analyzed a variety of parameters previously associated with the action of AID during the mutation reaction. Because of the importance of transcription for SHM, we measured IgL V region and *GFP* steady state transcript levels in the sequenced subclones by RT-PCR. This revealed that Bcl6^-/-^ subclones have levels of V region transcripts well above WT levels, perhaps because they have largely adopted a plasma cell state in which large quantities of antibody are produced. The other clones had V region transcript levels that were equivalent to (Pax5^R^ AID^R^ SD#10 clone 9) or within 2.5 fold of the levels seen in WT cells (Fig 4A), which is unlikely to explain the complete lack of V region GCV/SHM in the absence of Bcl6. Similarly, measurement of *GFP* expression revealed that while mRNA levels varied, one mutating WT clone (#6) expressed *GFP* comparably to the non-mutating clones (Fig 4B). We conclude that differences in V region or *GFP* expression levels do not account for the lack of mutation at these regions in the absence of Bcl6 (in Pax5^R^ AID^R^ SD#10 lines) or in the Bcl6-reconstituted lines.

AID is tightly regulated by nuclear import and export mechanisms that restrict its entry into the nucleus [32, 33]. To test the possibility that AID’s subcellular localization was altered in the absence of Bcl6, cytoplasmic and nuclear fractions from WT AID^O/E^ SD, Pax5^R^ AID^R^ SD#10 and Bcl6^R^ AID^R^ SD#10 DT40 cells were analyzed for AID protein. AID was clearly present in all nuclear fractions analyzed and Pax5 was specifically found in the nuclear fractions, demonstrating good cytoplasmic and nuclear partitioning (Fig 4C). Substantial amounts of AID levels were also detected in the cytoplasmic fractions. No consistent alteration in nuclear AID levels was observed that could be linked to the presence or absence of Bcl6 or that could explain the SHM defect.

It has been suggested that the V region is unusual in having a large amount of transcription-dependent single-stranded DNA when compared to other transcribed genes, and that this contributes to it being an efficient target for SHM [34]. To determine if Bcl6 was regulating the amount of single-stranded DNA at the IgL V region, sodium bisulfite sequencing was performed on unmanipulated (that is, not expressing ectopic AID) WT, Bcl6^-/-^, and Bcl6^-/-^ Pax5^R^ cells. A previous study found that generation of single-stranded DNA at a V region was independent of AID expression [34]. Notably, a higher frequency of single-stranded DNA patches was detected in the IgL V region in the non-mutating Bcl6^-/-^ and Bcl6^-/-^ Pax5^R^ lines as compared to the mutationally active WT cells (Fig 4D). We conclude that an inability to generate single-stranded DNA, as measured in this assay, is not responsible for the lack of V region SHM in the absence of Bcl6. However, it remains possible that AID’s ability to access the substrate is impaired.

### Spt5 association at the GFP reporter

**Fig 5:**
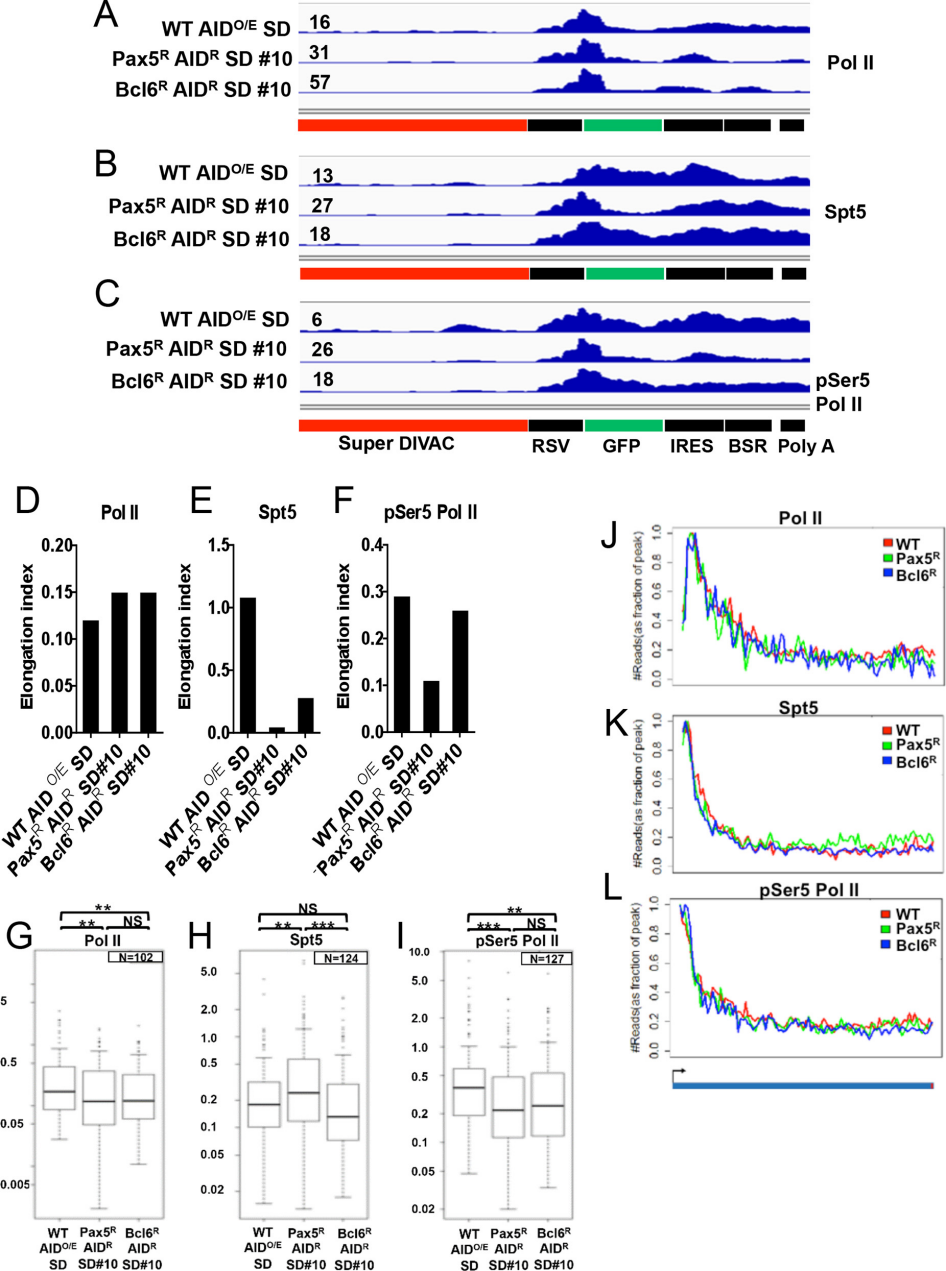
ChIP-seq analyses. ChIP-seq (reads per million mapped reads in raw (unfiltered) form) for A) RNA Pol II, B) Spt5, and C) RNA Pol II phosphorylated at serine 5 for the Super DIVAC GFP2 reporter construct in the cell lines indicated. The maximum value for the y-axis is indicated by the number to the left of each plot and was set to different values so as to keep all data within range and allow the shape of the data to be appreciated. D-F) Histogram plots of the A) Pol II, B) Spt5, and C) pSer5 Pol II elongation indexes for the indicated cell lines, calculated as the ChIP-seq signal (reads per million mapped reads per kb) within the *GFP* gene body divided by the signal surrounding the transcription start site (± 100 bp). G-I) Box plots showing the distribution of elongation indexes for approximately 100 highly expressed genes (chosen as described in Fig 5J-5L legend) for G) Pol II, H) Spt5, and I) pSer5 Pol II for the indicated cell lines. The lower and upper ends of the box denote the first and third quartiles respectively, with the horizontal bar indicating the median. Vertical bars define the minimum and maximum with outliers shown as dots. The signal surrounding the transcription start site for the *GFP* gene fell in the top 1% of signals for all genes for the three factors regardless of genetic background. Two tailed student t test was used to assess significant differences. **, p<0.01 and ***, p<0.001. J-L) Meta-analysis of the ChIP-seq signal for a selected group of highly occupied genes for each factor as indicated. Beginning from 100 bp upstream of the transcription site, the region of each gene from upstream of the transcription start site to the transcription termination site was divided into 100 bins and the reads per bp were calculated for each bin. The maximum number of reads was set to be 1, and other values were normalized accordingly. The plotted lines are the average values for the selected genes. Genes were selected by taking the top 200 genes based on strength of ChIP signals for the relevant factor in all three cell lines, and then retaining only those genes that overlapped among the top 200 in all three lines; Pol II N = 102, Spt5 N = 124, pSer5 Pol II N = 127.

Given recent data linking Spt5 to SHM (see Introduction), it was of interest to determine whether the recruitment of Spt5 to a mutation target was altered in the absence of Bcl6. We performed ChIP-seq for Spt5 in WT AID^O/E^ SD, Pax5^R^ AID^R^ SD#10, and Bcl6^R^ AID^R^ SD #10 using subclones previously analyzed for mutation by sequencing (Fig 3). We also performed ChIP-seq for total Pol II and Pol II phosphorylated at serine 5 (pSer5 Pol II), a modification that is associated with the initiating form of Pol II [35], to obtain a broader assessment of the transcriptional landscape in the presence and absence of Bcl6. Unfortunately, pilot experiments with DT40 cells lacking the rearranged IgL V region (IgL^-^ cells) [29] yielded many reads that mapped to the rearranged V region (data not shown), likely stemming from pseudo V elements and the V region of the un-rearranged allele. Hence, we focused our analysis on the *GFP* transcription unit in the Super DIVAC GFP2 reporter (Fig 5A–5C). For quantitative analysis of the ChIP-Seq profiles, we calculated “elongation indexes”, which were defined as the Pol II (or other protein of interest) ChIP signal within the gene body divided by the signal surrounding the transcription start site (± 100 bp) (Fig 5C–5E). The elongation index normalizes for differences in the overall strength of signal routinely obtained in different ChIP-seq experiments (note different y-axis scales in the data of Fig 5A–5C).

Total Pol II was detected in the three cell lines as a prominent peak near the 3' end of the rous sarcoma virus (RSV) promoter, with a sharp decrease in signal in the first 100 bp of the GFP gene, with the signal remaining low throughout the GFP coding region (Fig 5A). Such a Pol II profile is typical of many transcribed genes [36]. No gross differences in the ChIP-seq profiles were observed among the three lines and consistent with this, the Pol II elongation indexes were not dramatically different for the three cell lines, with the amount of Pol II associated with the gene body relative to the promoter increased slightly in the Pax5-reconstituted and Bcl6-reconstituted cells relative to WT (Fig 5D).

Spt5 recruitment was readily detected at the promoter region in the three cell lines analyzed, but striking differences were visible in the *GFP* gene body (Fig 5B). In WT cells, Spt5 association remained high throughout *GFP*, while in the absence of Bcl6 (Pax5^R^ AID^R^ SD #10), Spt5 levels abruptly decreased near the beginning of *GFP* and remained low through the remainder of the gene (Fig 5B). The Bcl6-reconstitued cells (Bcl6^R^ AID^R^ SD #10) exhibited a substantial signal for Spt5 extending through most of the *GFP* coding region (Fig 5B). The elongation indexes reflected these profiles, with a nearly 22-fold drop in the Bcl6-deficient cells relative to the WT cells (Fig 5E), indicating a large decrease in Spt5 levels in the gene body relative to the promoter as a result of the loss of Bcl6. The Spt5 elongation index in the Bcl6-reconstituted cells was 5.5 fold above that in the Bcl6-deficient cells but did not reach that of WT cells, perhaps due to incomplete Bcl6 reconstitution (Fig 5E). These data suggest that Spt5 association with the *GFP* gene body is selectively increased, relative to the promoter, in a Bcl6-dependent manner. We note that Spt5 ChIP signals increased downstream of *GFP* in the Pax5 reconstituted cells to levels roughly comparable to those in the other cell lines tested. While we do not have an explanation for the increased Spt5 signal downstream of *GFP*, the IRES element present in this location adopts substantial structure at the RNA level [37] which could conceivably affect transcription parameters.

The pSer5 Pol II profiles qualitatively resembled those for Spt5, with a decrease in levels observed in the *GFP* gene body relative to the promoter in the Bcl6-deficient cells compared to the other two cell lines (Fig 5C). This was reflected by a lower pSer5 Pol II elongation index in the Bcl6-deficient cells than the other two lines, although the decrease was not as strong as for Spt5 (Fig 5F). Taken together, the data suggest that Bcl6 promotes the association of Spt5 and, to a lesser extent, pSer5 Pol II with the *GFP* gene body.

To determine whether the large decrease in the Spt5 elongation index in Bcl6-deficient versus WT cells was specific for the *GFP* transcription unit, we calculated the elongation index for Pol II, Spt5, and pSer5 Pol II for approximately 100 endogenous genes with the strongest ChIP-seq signals (see Methods and Fig 5G–5I), an appropriate comparison group because the GFP2 reporter also exhibited some of the strongest ChIP-seq signals for these factors. This analysis revealed small but significant variations in the average elongation index among the cell lines for the three factors examined (Fig 5G–5I). The interpretation of these differences or how they might relate to the presence or absence of Bcl6 is currently unclear. Importantly, the Spt5 elongation index for the control genes was higher in the Bcl6-deficient cells than the other two lines (Fig 5H), the opposite of what was observed for the GFP2 reporter (Fig 5E). This indicates that the strong relative reduction in Spt5 association with the *GFP* gene body in the absence of Bcl6 is not due to a global reduction in Spt5 recruitment to gene bodies relative to promoter regions. We also note that the large (22-fold) difference in the Spt5 elongation index for the *GFP* gene between WT and Bcl6-deficient cells is due to a combination of a particularly high elongation index of ~1.1 in the WT cells (top 5% of the values obtained from WT cells for the 124 genes analyzed in Fig 5H) and a low elongation index of ~0.05 in Bcl6-deficient cells (bottom 7% of the values obtained for the 124 genes in Bcl6-deficient cells).

We also performed a meta-analysis to generate the profile for Pol II, Spt5, and pSer5 Pol II across these same groups of endogenous genes, generating the average (normalized) levels of reads for each factor from just upstream of the transcription start site to the transcription termination site. This revealed similar profiles for the three factors in the cell lines analyzed (Fig 5J–5L), with a rapid decline in ChIP signal observed with increased distance from transcription start site. In particular, this analysis emphasizes that a sharp decrease in Spt5 signals after the transcription start site is the typical profile for highly expressed genes (Fig 5K). Such a profile stands in stark contrast to the Spt5 profile seen at the *GFP* transcription unit in WT DT40 cells (Fig 5B), in which Spt5 levels remain high throughout the *GFP* gene. Taken together, the data of Fig 5 suggest that Bcl6 function in DT40 cells results in an increased association of Spt5 with a SHM target region in a manner that is not seen at most other highly expressed genes.

## Discussion

### Bcl6 is required for SHM and GCV

Our work demonstrates that Bcl6 is required for detectable SHM and GCV in DT40 cells. Robust expression and nuclear accumulation of AID protein was unable to induce any mutation above background levels in the absence of Bcl6. Importantly, this SHM defect exists despite a well transcribed V region where single-stranded DNA, the AID substrate, is efficiently generated. Additionally, an artificial GFP transgene, when transcribed in the presence of a potent DIVAC element, was also unable to be mutated in the absence of Bcl6, even after prolonged culture of the cells. The *GFP* transcription unit utilizes a distinct viral promoter and DNA substrate, and its failure to mutate in the absence of Bcl6 indicates that the SHM defect is not restricted to the IgL V region and its promoter. Our findings build on those previously reported [26] by demonstrating that the Bcl6-controlled differentiation and gene expression program potentiates SHM/GCV in DT40 cells by a mechanism distinct from, and in addition to, its role in enabling AID expression. However, our data cannot distinguish between a fairly direct role for Bcl6 in activating SHM/GCV or a less direct role where Bcl6 establishes a physiological or developmental state which is permissive for SHM/GCV.

While the results of the bisulfite assay suggested efficient generation of transcription-associated single-stranded DNA in the absence of Bcl6, it remains unknown whether this assay measures the substrate that AID actually acts on. Indeed, our data demonstrate that the presence of WT levels of the single-stranded DNA patches detected by this assay are not sufficient, together with robust AID expression and a strong DIVAC element, to allow for detectable SHM in cells lacking Bcl6. In this regard, our data do not address the question of whether AID can gain access to its substrate in the absence of Bcl6. Phosphorylation of serine 38 on AID is important for its interaction with replication protein A [38] and induction of SHM [39], and it is plausible that this interaction is important for AID to gain access to the single-stranded DNA made available during transcription. We were unsuccessful in our efforts to determine whether S38 phosphorylation of AID is deficient in the absence of Bcl6. Furthermore, despite extensive efforts, we have not been able to detect AID binding at the IgL V region or GFP2 cassette by ChIP in a variety of mutationally active DT40 lines. Hence, it remains unknown whether Bcl6 contributes to AID recruitment to mutation substrates.

We considered the possibility that the SD#10 GFP2 reporter integration site was intrinsically resistant to SHM, and while this cannot be ruled out, it is unlikely for several reasons. *GFP* transcription from the SD#10 integration site was robust (e.g., Fig 3A), suggesting integration into an “open”, transcriptionally permissive chromatin landscape that should be accessible to AID. Furthermore, it has been previously shown that a DIVAC GFP reporter can be integrated at a variety of locations in the DT40 genome and still be robustly targeted for SHM [29]. Consistent with this, all 11 random Super DIVAC GFP2 reporter integration sites tested in WT AID^O/E^ subclones exhibited readily detectable GFP loss and 10 of 11 underwent robust SHM (Fig 2C). We also note that the SD#10 GFP2 reporter integration exhibited a mutation phenotype almost identical to that of the IgL V region (Fig 3C and 3D). We conclude that an intrinsic problem with the SD#10 integration site is not likely to explain the SHM defect in Bcl6-deficient DT40 cells.

We also considered the possibility that, in the absence of Bcl6, AID deamination events in the IgL V region and GFP occur but are repaired in an error-free manner, preventing their detection. This is extremely unlikely given that *Ung* expression is strongly reduced in the absence of Bcl6 and that a reduction in Ung activity in DT40 cells actually potentiates the detection of AID-mediated point mutations, presumably by reducing high-fidelity repair pathways [27, 28].

Our efforts to confirm the link between the absence of Bcl6 and the defect in SHM/GCV through Bcl6 re-expression and full reconstitution of the Bcl6 gene expression program were not successful. Using the same Bcl6-deficient line, a previous study described the generation of one clone that was successfully reconstituted with Bcl6 [26]. Importantly, this same study demonstrated that loss of Bcl6 protein expression in DT40 cells leads to a transition into a plasma cell state marked by high Blimp1 expression [26], consistent with our own observations. Blimp1 is a well known repressor of Bcl6 gene expression [40, 41]. Hence, our efforts to re-establish Bcl6 expression were in the context of cells in which the plasma cell gene program was highly active and could inhibit Bcl6 expression and/or function and, thereby, inhibit SHM/GCV [42, 43]. This might help explain our difficulty in establishing a robust Bcl6 reconstitution. Alternatively, it is possible that a plasma cell state precludes SHM/GCV independently of its effect on Bcl6 expression and/or function. Our data do not distinguish between these two models.

### Bcl6 and the mechanism of SHM

We did not detect an effect of Bcl6 on AID subcellular compartmentalization. However, recent work has demonstrated that AID undergoes maturation steps in the cytoplasm before it is imported into the nucleus as a fully functional protein. For example, interactions with heat-shock protein 90 and eukaryotic elongation factor 1 α were found to produce and sequester active AID protein in the cytoplasm [44]. It is possible that Bcl6-deficiency disrupts an AID maturation pathway resulting in poor AID activity in the nucleus.

Given the numerous links identified between SHM and transcription, it was important to compare the transcriptional landscape at the mutation reporter construct in Bcl6-sufficient and–deficient cells. We found that strong total Pol II and pSer5 Pol II signals were present over the transcription start site independent of the presence of Bcl6. Normally, RNA Pol II converts from an initiating to an elongating form within 100 bp of the start of transcription [35]. Interestingly, the WT and Bcl6-reconstituted cells demonstrated a continued association of the pSer5 Pol II modification with the body of the *GFP* gene to a greater extent than in the Bcl6-deficient Pax5-reconstituted cells. Hence, the data suggest that retention of pSer5 Pol II in the *GFP* gene body is increased by Bcl6. Intriguingly, our prior study found a significant two-fold increase in pSer5 Pol II in the *GFP* gene body in the presence of DIVAC as compared to its absence [16]. This raises the possibility that there is a mechanistic link between Bcl6 and the function of DIVAC, although we note that the restoration of pSer5 Pol II in the *GFP* gene body that accompanied reconstitution of Bcl6 was not sufficient to allow for detectable mutation.

We also observed, in both Bcl6-sufficient and -deficient settings, prominent Spt5 peaks surrounding the transcription start site of the GFP2 reporter, demonstrating that Spt5 recruitment to the promoter does not require Bcl6. In contrast, association of Spt5 with the *GFP* gene body was strongly reduced in the Bcl6-deficient versus WT context, with the Bcl6-reconstituted cells exhibiting an intermediate phenotype. Our data support a Bcl6-dependent retention of Spt5 in the mutation target region, which could contribute to AID targeting and SHM/GCV. However, the intermediate level of Spt5 recruitment to the *GFP* gene body seen in the Bcl6-reconstituted cells was not sufficient to allow for detectable SHM. We cannot rule out the possibility that a threshold level of Spt5 is important for AID activity and SHM and that this threshold was not reached in the Bcl6-reconstituted cells. Notably, our previous study did not observe a difference in Spt5 levels in the *GFP* gene body in the presence versus the absence of DIVAC [16], suggesting that the activation of a gene for SHM involves more than the recruitment of Spt5. We are currently performing a comprehensive analysis to determine how the presence of DIVAC alters the transcriptional parameters of the mutation cassette.

A link between Spt5 and SHM was supported by findings that Spt5 interacts with AID and that its binding in the genome correlates with sites of SHM [25]. This link was recently extended with the observation that Spt5 associated with an Igh V region in germinal center B cells (which undergo SHM) but not with the same V region in *ex vivo* activated B cells [45], which were thought not to undergo V region SHM [46]. A very recent study, however, finds that *ex vivo* activated B cells do in fact perform IgH V region SHM in an AID hot-spot focused manner similar to that of germinal center B cells that have accumulated a small number of mutations (presumably because they had engaged in only a few cycles of SHM) [47]. These results emphasize that the connection between SHM and transcription cannot be reduced to a question of levels of Spt5 recruitment. Indeed, our data indicate that substantial Spt5 association with the *GFP* target gene (as in the Bcl6-reconstituted cells) was unable to activate detectable SHM without a complete, Bcl6-dependent, gene expression program. Based on our findings, we favor a model where Spt5 (in a Bcl6-dependent manner) associates with transcribed SHM target genes enriched in stalled RNA Pol II and AID, and that by virtue of its interaction with Spt5 (among other factors), AID gains prolonged access to single-stranded DNA to initiate SHM.

In summary, our data establish a previously unknown role for the Bcl6-controlled differentiation and gene expression program in the mechanism of SHM. We argue that this is accomplished, at least in part, through Bcl6-dependent association of Spt5 with SHM substrates.

## Materials and Methods

### Cell culture

DT40 cells [26] were grown at 41°C with 5% CO_2_ in RPMI-1640 (Lonza) with additional supplements: 10% fetal bovine serum (Lonza), 1% chicken serum (Sigma), 2 mM L-glutamine, penicillin/streptomycin, and 0.1 mM β-mercaptoethanol. Generation of Bcl6^-/-^, Bcl6^-/-^ Pax5^R^, and Bcl6^-/-^ Bcl6^R^ cells was described previously [26]. Drug selection markers were deleted from these three lines by treating 3×10^5^ cells with 6 μM his-Tat-NLS-Cre recombinant protein [48] for 1 hour in 300 μl serum-free media in the incubator. Cells were single-cell seeded after the incubation, and drug sensitive clones were identified 14–17 days later. WT AID^O/E^, Bcl6^-/-^ AID^R^, and Bcl6^-/-^Pax5^R^ AID^R^ cells were generated by infection with an MSCV-IRES-thy1.1 retroviral expression vector with the chicken AID cDNA cloned between Bgl II and Not I restriction sites. Bcl6^-/-^Pax5^R^ AID^R^ Bcl6^R^ lines were generated by transfection of the pLox puro expression vector containing an N-terminally 3X Flag tagged chicken Bcl6 cDNA [26] and subsequently infected with an MSCV-IRES-neomycin retroviral vector also containing an N-terminally 3X Flag tagged chicken Bcl6 cDNA cloned via In-Fusion HD Cloning (Clontech). Stable integrates of these constructs were selected with either 1 μg/mL puromycin or 2 mg/mL G418 for neomycin selection.

### Retroviral infections

293T cells [49] were co-transfected with the pKAT2 retroviral packaging plasmid and MSCV-IRES-viral expression plasmid of interest using the calcium phosphate method. Two days post transfection, viral supernatant was harvested and added to DT40 cells, followed by centrifugation at room temperature, 1800 rpm, for one hour. An equal volume of fresh DT40 media was then added to the cells.

### Transfection of GFP reporter constructs

Stable cell lines expressing the GFP2 cassette (with or without Super DIVAC) were made by electroporating 75μg Not I-linearized pIgL^-^ GFP2 vector [29] at 25 μF and 700 V (Bio-Rad Gene Pulser). Transfectants were selected with 15 μg/mL blasticidin (Invitrogen).

### Flow cytometry

For the AID reconstitution experiments, DT40 cells were stained in PBS with anti-thy1.1 antibody and thy 1.1 positive cells were purified by cell sorting. Subclones were evaluated for GFP expression via flow cytometry (FACSCalibur, BD Biosciences). Samples were first gated by FSC (forward-scatter) and SSC (side-scattered) for live cells followed by creation of a GFP negative gate drawn one log below the primary GFP+ population (Flow Jo software). GFP loss values above 50% were excluded from the fluctuation analysis to prevent inclusion of any cells that were GFP-negative at the time of subcloning.

### Antibodies

The antibodies used were: for flow cytometry, APC conjugated anti-thy1.1 (BD Pharmingen #561409); for Western Blot, anti-AID (Invitrogen #39–2500), anti-Pax5 (Santa Cruz # sc-13146), anti-β-actin (Sigma Aldrich # A5441), anti-Flag (Sigma Aldrich #1804), Rabbit anti-mouse HRP Fcγ specific (Jackson Immunology #315-001-008); for ChIP Seq, RNA Pol II (Santa Cruz, #sc-899x); RNA Pol II CTD PhosphoS5 (Abcam, #ab5131), and Spt5 (Santa Cruz #sc28678).

### Western Blot Analysis

Cells were sonicated and lysed in 0.1% Triton-X buffer [50 mM Tris, pH 7.4; 150 mM NaCl; 1mM sodium orthovanadate; 25mM β-glycerophophate, plus protease and phosphatase inhibitor cocktail (Thermo Scientific #78443)] and electrophoresed on SDS-PAGE gels following protein quantitation and equivalent loading for each lane. The membrane was blotted with the appropriate primary and secondary antibodies and developed using enhanced chemiluminescence reagents (GE-Amersham).

### Mutation sequencing procedure

3x10^6^ DT40 cells were collected and washed in PBS, resuspended in 400 μL lysis buffer (100 mM Tris pH 8.0, 5 mM EDTA, 0.1M NaCl, 0.6% SDS, and 600 μg of proteinase K) and incubated overnight at 55°C. DNA was purified via phenol/chloroform extraction followed by precipitation in 100% ethanol. The IgL V region and *GFP* genes were amplified from approximately 100–200 ng of genomic DNA using Phusion Polymerase (NEB) and company specified parameters for 30 cycles. PCR fragments electrophoresed on an agarose gel and gel extracted using QIAquick Gel Extraction Kit (Qiagen) with adenine overhangs added to the PCR fragments. PCR fragments were cloned into the pCR2.1-TOPO vector from the TOPO TA cloning kit (Invitrogen) and transformed into Max Efficiency DH5α-T1R chemically competent cells (Invitrogen) with X-gal added to bacterial plates for blue/white colony screening. Selected colonies were purified and sequenced at Beckman Coulter genomics using the M13 forward and reverse primers.

### Mutation sequencing analysis

Sequences were analyzed for mutation in the Sequencher software platform by simultaneously comparing all sequences from an individual sub-clone. Point mutations harbored in more than one sequence at an identical position were considered clonally related and only counted one time as a mutation. In the case of gene conversion events, only stretches of DNA that exactly matched at least one pseudo V element in the Pub Med chicken genome database was considered a gene conversion event. Mutation event frequencies were calculated by dividing the number of observed mutation events by the total number of base pairs sequenced.

### RT-PCR analyses

Total RNA was isolated from each DT40 clone with the RNeasy Mini Kit (Qiagen), with 1 μg treated with DNase I (Invitrogen) and revere transcribed by Superscript II reverse transcriptase (Qiagen) using random primers (Invitrogen). Duplicate PCR reactions were performed with SYBR Green master mix (Bio-Rad) using company specified cycling parameters. cDNA values were normalized to Rab7a expression levels.

### Primers

Primers sets used for RT-PCR analyses were: A*icda* (forward ACACCGTCTGAAACCCAG, reverse AGAAACGTGGAATGCTCTGTAC); *Bach2* (forward GCCAGTCTCTCCCCAGCTCTC, reverse GCTGGAGGTCCTCGTTCTGGT); *Bcl6* (forward GAGAAGCCATACCCCTGTGA, reverse TGCACCTTGGTGTTTGTGAT); *GFP* (forward TGACCCTGAAGTTCATCTGC, reverse GAAGTCGTGCTGCTTCATGT) *Mitf* (forward GGACTGTCCCTTGTTCCATCC, reverse CCGAGGTTGTCACTGAAGGTG); *Prdm1* (forward ACACAGCGGAGAGAGACCAT, reverse GCACAGCTTGCACTGGTAAG); *Rab7a* (forward GCCCCTAACACATTCAAAACC, reverse GCTTGTGCCCGTTTTGTG); *Ung* (forward ATGGGGTTGTTTTCATGCTGTG, reverse GCAGCTCGTTTGTCTTGGAGAA); V region (forward CCGATGACGAGGCTGTCTA, reverse AGGACGGTCAGGGTTGTC).

Primer sets used for all sequencing based analyses were: *GFP* (forward TCCTTCAGCCCCTTGTTG, reverse TAAACGCCATTTGACC); V region (forward CCATGGCCTGGGCTCCTCTCCTCCTG, reverse GACAGCACTTACCTGGACAGCTG).

### Cytoplasmic and nuclear fractionation

DT40 cells were washed twice with ice cold PBS and treated with 500 μL of 1X Hypotonic buffer (20 mM Tris-HCl, pH 7.4 10 mM NaCl, 3 mM MgCl_2_) for 15 minutes on ice. 25 μL of 10% NP40 detergent was added to the sample before vortexing for 10 seconds. Samples were spun at 3000 rpm for ten minutes at 4° to isolate the cytoplasmic fraction. The nuclear pellet was washed 3X with hypotonic buffer, resuspended in Cell Extraction Buffer (Invitrogen), and sonicated for 5 minutes (30 seconds on/off). Both cytoplasmic and nuclear fractions were quantitated before loading equivalent amounts for western blot analysis.

### Sodium bisulfite analysis

Sodium bisulfite experiments were performed as previously described [34] on 10^7^ DT40 cells. The 5' and 3' ends of a patch were defined by the first and last unmodified C (top strand) and the first and last unmodified G (bottom strand) flanking at least two successive transition mutations (patch minimum). The total number of patches divided by the number of base pairs sequenced was calculated to give the % single-stranded patches.

### ChIP-seq sample preparation

40x10^6^ cells were harvested and cross-linked with 1% formaldehyde for 10 minutes at room temperature and then quenched with 0.125 M glycine. The cross-linked cells were lysed in 600 μL RIPA buffer (10mM Tris, 1mM EDTA, 1% Triton X-100, 0.1% sodium deoxycholate and 0.1% SDS) containing 0.8 M NaCl and chromatin sheared to a mean size of 150–200 bp. Overnight incubation of the sheared chromatin with antibody (5–7 μg) preceded the addition of Protein G Dynabeads (Invitrogen) to collect immune complexes. The immune complexes were washed under the following conditions: 2X with low salt wash buffer (20 mM Tris HCl pH 8.0; 150 mM NaCl, 2 mM EDTA, 1.0% Triton X-100, 0.1%SDS), 2X with RIPA buffer, 1X with TE + 50 mM NaCl, 1X with TE. To reverse all crosslinks and elute DNA, 100 μL of TE was added along with 5 μL of 20 mg/mL proteinase K for overnight digestion. ChIP samples were then phenol chloroform extracted and ethanol precipitated in 100% ethanol. Samples were prepared for Illumina Next Seq 500 sequencing using the TruSeq ChIP sample preparation kit (Illumina).

### ChIP-seq computational analyses

General ChIP-seq analysis was performed as follows: The 76 bp reads obtained after the sequencing run were trimmed using fastq_trimmer program from fastx_toolkit-0.0.13 (5 bp from 5' and 20 bp from 3') to get 50 bp reads for alignment. The reads were aligned to chicken genome (build galGal4) using bowtie 0.12.7 allowing 2 mismatches out of 50 and removing all non-uniquely aligned reads. To align plasmid-derived reads, we added the plasmid sequence to the chicken genome as a separate component. The aligned reads were further filtered such that only non-redundant reads were used. Wig files were generated using MACS-1.4.2 with parameter-space 100, and then normalized to reads per million (RPM). Scores in the wig files represent the RPM over overlapping 100 bp window.

The calculation of elongation indexes for the GFP reporter construct was performed as follows: a promoter proximal region was defined as (-100)-(+100) bp from the transcription start site (TSS) and the promoter distal region was defined as the region (+101) bp from the transcription start site to the end of the GFP coding region. The ratio between the signal obtained within the gene body and promoter proximal region defined the elongation index for the GFP cassette.

A similar analysis was performed with chicken refSeq genes. Since a typical gene is larger than GFP (~7 kb vs 700 bp), we extended the promoter proximal region and defined it as (-100)-(+300) bp from the TSS. Accordingly, the promoter distal was defined as (+300) bp from the transcription start site to the transcription termination site.

### Statistical Analyses

A Student t test was used to assess the significance of Bcl6-dependent gene expression data (one tailed) and genome-wide elongation index data (two-tailed). A z-test, which compares proportions of two independent groups, was used to assess significance of mutation frequencies. A Mann-Whitney test was used to compare the median GFP loss values for significant differences in the GFP loss assay.

## Supporting Information

S1 Fig Efficacy of the Bcl6 reconstitution. A) RT-PCR analysis for Bcl6 mRNA expression in the indicated cell lines. Data are normalized to expression levels of the housekeeping gene Rab7a. ND, not detected. B) Anti-Flag western blot for Bcl6 protein in cytoplasmic and nuclear fractions for the indicated cell lines. Cytoplasmic and nuclear fractions were quantitated and equivalent amounts of protein were loaded. *, unknown bands. C-G) RT-PCR analysis of Bcl6 target gene RNA levels in the indicated cell lines. Data are presented as the average of the signal obtained from four independent RNA preparations (two independent extractions per subclone assayed) after normalization to the signal obtained for Rab7a. Error bars represent the standard error of the mean (SEM). One tailed student t test used to assess the significance of gene expression changes. *, p<0.05; **, p<0.01; ***, p<0.001 and NS, not significant.
(TIF)

S1 Table ChIP-seq read information. Read number data for the ChIP-seq analyses.
(PDF)
