# Supplementary figures and images for: Bcl6 Is Required for Somatic Hypermutation and Gene Conversion in Chicken DT40 Cells
Source: PLoS One. 2016 Feb 22;11(2):e0149146. doi: 10.1371/journal.pone.0149146 (PMC4762950; doi:10.1371/journal.pone.0149146)

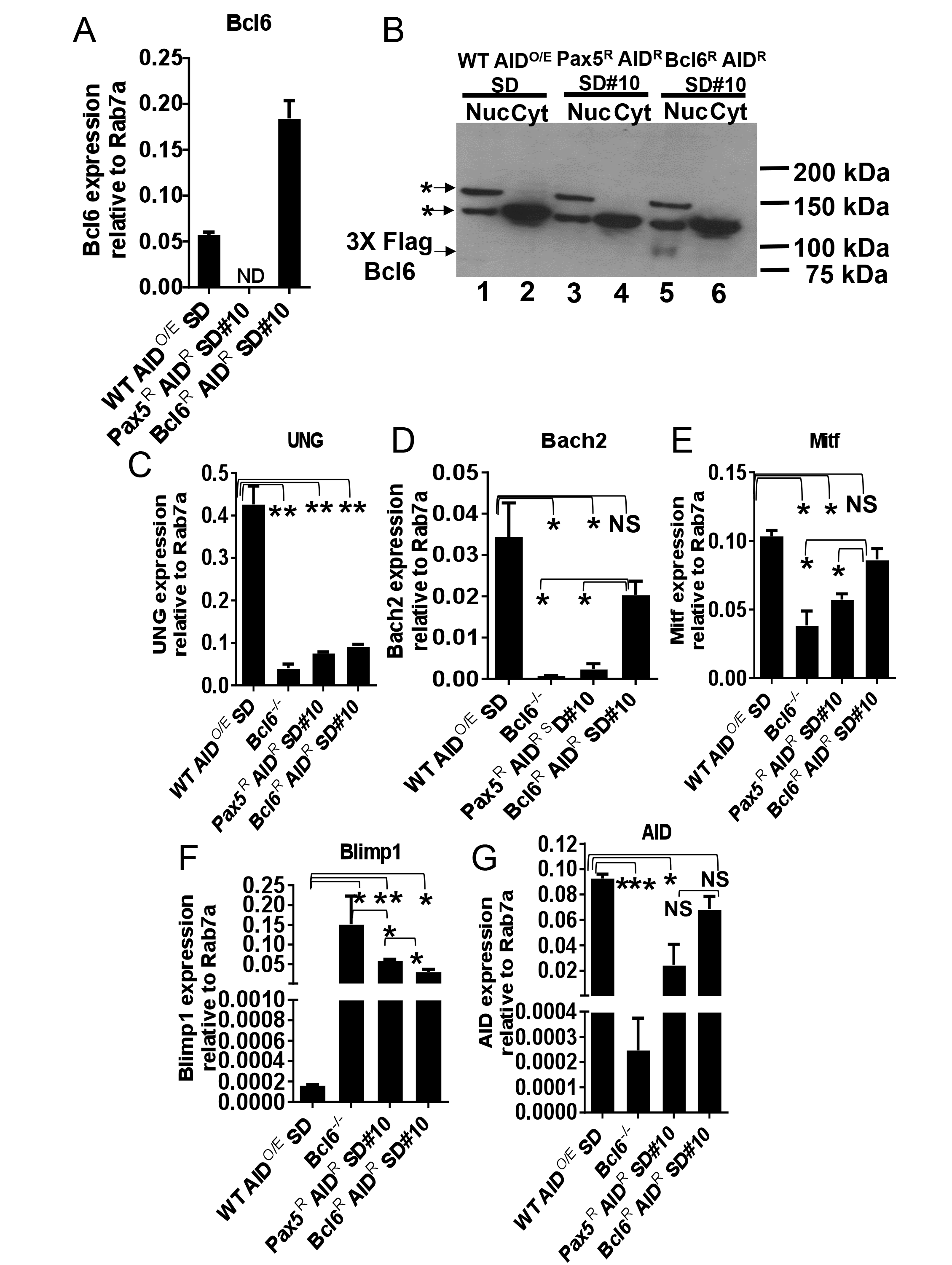

Supplement: S1 Fig — A) RT-PCR analysis for Bcl6 mRNA expression in the indicated cell lines. Data are normalized to expression levels of the housekeeping gene Rab7a. ND, not detected. B) Anti-Flag western blot for Bcl6 protein in cytoplasmic and nuclear fractions for the indicated cell lines. Cytoplasmic and nuclear fractions were quantitated and equivalent amounts of protein were loaded. *, unknown bands. C-G) RT-PCR analysis of Bcl6 target gene RNA levels in the indicated cell lines. Data are presented as the average of the signal obtained from four independent RNA preparations (two independent extractions per subclone assayed) after normalization to the signal obtained for Rab7a. Error bars represent the standard error of the mean (SEM). One tailed student t test used to assess the significance of gene expression changes. *, p<0.05; **, p<0.01; ***, p<0.001 and NS, not significant. (TIF) [file pone.0149146.s001.tif]
