# Supplementary material for: Bcl6 Is Required for Somatic Hypermutation and Gene Conversion in Chicken DT40 Cells
Source: PLoS One. 2016 Feb 22;11(2):e0149146. doi: 10.1371/journal.pone.0149146 (PMC4762950; doi:10.1371/journal.pone.0149146)
Supplement: S1 Table — Read number data for the ChIP-seq analyses. (PDF) [file pone.0149146.s002.pdf]

Table S1. ChIP-seq read information

Cell type	Genotype	IP	# Total reads	Reference Genome	# Uniquely aligned reads	# Uniquely aligned, non-redundant reads
DT40	WT $AID^{O/E}$ SD	Pol II	107,768,440	galGal4	51,673,885	3,920,025
DT40	WT $AID^{O/E}$ SD	Spt5	159,889,769	galGal4	71,601,661	2,274,165
DT40	WT $AID^{O/E}$ SD	pSer5 Pol II	74,184,372	galGal4	35,742,238	7,342,490
DT40	$Pax5^R$ AID^R SD#10	Pol II	38,811,452	galGal4	18,551,053	441,196
DT40	$Pax5^R$ AID^R SD#10	Spt5	40,651,148	galGal4	18,474,952	1,077,649
DT40	$Pax5^R$ AID^R SD#10	pSer5 Pol II	133,382,768	galGal4	64,675,004	1,170,761
DT40	$Bcl6^R$ AID^R SD#10	Pol II	29,139,092	galGal4	13,489,788	299,203
DT40	$Bcl6^R$ AID^R SD#10	Spt5	89,800,846	galGal4	43,060,008	3,774,591
DT40	$Bcl6^R$ AID^R SD#10	pSer5 Pol II	117,683,921	galGal4	56,482,649	2,651,265
